# Comprehensive knowledge on cervical cancer, attitude towards its screening and associated factors among women aged 30–49 years in Finote Selam town, northwest Ethiopia

**DOI:** 10.1186/s12978-018-0471-1

**Published:** 2018-02-14

**Authors:** Alehegn Bishaw Geremew, Abebaw Addis Gelagay, Telake Azale

**Affiliations:** 1 Department of Reproductive Health, Institute of Public Health, College of Medicine and Health Science, University of Gondar, 196 Gondar, Ethiopia; 2 Department of Health Education and Behavioral Sciences, Institute of Public Health, College of Medicine and Health Sciences, University of Gondar, Gondar, Ethiopia

**Keywords:** Women aged 30–49 years, Knowledge, Attitude, Ethiopia

## Abstract

**Background:**

Screening services for cervical pre-cancerous lesions is currently available for all women aged 30–49 years at public hospitals in Ethiopia. Though women’s knowledge and their attitude are determinants for the uptake the screening service, there is limited information on these regards. Therefore, this study aimed to assess comprehensive knowledge on cervical cancer, attitudes towards the screening, and associated factors among women aged 30–49 years at Finote Selam town, northwest Ethiopia.

**Methods:**

A community based cross-sectional study was conducted from March 30, to April 15, 2017. The sample size calculated for this study was 1224 and a cluster sampling technique was used to select the participants from three randomly selected kebeles. Epi-Info version 7 and Statistical Package for Social Sciences version 20 were used for data entry and analysis respectively. A binary logistic regression model was used. In multivariable logistic analysis, adjusted odds ratio with a 95% confidence interval was used to determine the presence and strength of associations between covariate and outcome variable.

**Results:**

A total of 1137 women participated in this study. Nearly one third, 30.3% (95%CI: 27.7, 32.9) of the women had knowledge of cervical cancer, and 58.1% (95% CI: 55, 62.2) had a favorable attitude towards cervical cancer screening. In the multivariable analysis, having college and above education (AOR = 7.21, 95%CI: 3.41, 15.29), knowing someone with cervical cancer (AOR =5.38, 95%CI: 2.38, 12.15), and having a history of sexually transmitted diseases (AOR = 2.75, 95%CI: 1.24, 6.04) were significantly associated with knowledge on cervical cancer. Meanwhile, college and above educational status (AOR = 2.56, 95%CI: 1.14, 5.69), knowing someone with cervical cancer (AOR = 3.24, 95%CI: 1.14, 9.15), and having knowledge of cervical cancer (AOR = 3, 95%CI: 1.97, 4.29) were positively associated with favorable attitudes towards cervical cancer screening.

**Conclusion:**

The proportion of women who had knowledge on cervical cancer was low where as relatively, a large proportion of the study participants in this study had favorable attitude towards cervical cancer screening. Educational status, knowing someone with cervical cancer, a history of sexually transmitted diseases was factors affecting both women’s knowledge and their attitude. Having knowledge on cervical cancer was factor affecting attitude towards screening services. Provision of information, education, and counseling about the disease and screening service are mandatory to address their knowledge gap and to improve women’s attitude towards screening service.

## Plain English summary

Screening services for cervical pre-cancerous lesions are currently available for all women aged 30–49 years at public hospitals in Ethiopia. Though women’s knowledge and their attitude are determinants for the uptake the screening service, there is limited information on these. Therefore, this study aimed to assess the comprehensive knowledge of cervical cancer, attitudes towards the screening, and associated factors among women aged 30–49 years at Finote Selam town, northwest Ethiopia.

A total of 1137 women participated in this study. Nearly one third, 30.3% of the women had comprehensive knowledge on cervical cancer, and 58.1% had a favorable attitude towards cervical cancer screening. Having college and above education, knowing someone with cervical cancer, and having a history of sexually transmitted diseases were significantly associated with comprehensive knowledge of cervical cancer. Meanwhile, college and above educational status, knowing someone with cervical cancer, and having comprehensive knowledge of cervical cancer were positively associated with favorable attitude towards cervical cancer screening. Therefore we recommended that provision of information, education, and counseling about the disease and screening service are mandatory to address the knowledge gap and to improve women’s attitude towards screening services.

## Background

Despite the fact that cervical cancer is preventable, it is reported that there were 485,000 cervical cancer cases and 236,000 deaths of women due to cervical cancer in 2013 worldwide [1]. Almost 87% of the deaths occurred in less developed regions, it was the second most commonly diagnosed cancer and the third leading cause of cancer death among females in less developed countries [2].

East Africa has the highest sub- regional incidence of cervical cancer in which the age standardised rate is 42.7 per 100,000 women, followed by Southern Africa with 31.5 per 100,000 women [3]. In Ethiopia, an estimated 7095 new cervical cancer cases were diagnosed, and 4732 die annually from it in 2012 [4]. New cases of cancer were diagnosed in the Black Lion specialized Hospital data set between 1997 and 2012, and the result revealed that 31.8% were cervical cancer patients [5].

The large geographic variations in cervical cancer rates reflect the presence of differences in the accessibility of screening service because the presence of screening service detects precancerous lesion and helps early initiation of treatment of the lesion before it progresses to cancer stage [6]. A population survey indicates the average cervical cancer screening in developing countries is 19%while it is 63% in developed countries and ranging from 1% to 73%respectively [7]. A meta-analysis noted that the risk of dying from cervical cancer was 35% lower among women invited to screening with cytology testing than among women who were not offered screening services [8].

Having knowledge on cervical cancer and it’s screening is associated with the uptake of services [9], favourable attitudes towards cervical cancer screening is also associated with the uptake of screening services [10, 11]. Cervical cancer screening helps to detect pre-cancerous lesions before it advances to a cancerous stage which in turn reduces its related mortality rates. In Ethiopia, screening services for cervical pre-cancerous lesion is available for all women aged 30–49 years at public health institutions [12]. Women’s knowledge on cervical cancer and their attitude for screening determine the uptake of service [13]. However, evidence on women’s knowledge of cervical cancer and their attitude towards screening at the community level is very limited in Ethiopia. Therefore, a community based study was conducted to assess knowledge of cervical cancer, attitude towards its screening and associated factors among women aged 30–49 years at Finote Selam town, northwest Ethiopia.

## Methods

### Study design and setting

A community based cross-sectional study was conducted from March 30, to April 15, 2017 at Finote Selam town, northwest Ethiopia. Finote Selam town is located in West Gojam Administration Zone of the Amhara Regional State, northwest Ethiopia. According to the population projection of Ethiopia for all regions at woreda level 2017, the total population of the town is estimated to be 38,399. Out of these, 19,923 are male and 18,476 female [14]. At the moment, the town has five kebeles, the smallest administrative units. The total number of households was 5530.

Finote Selam town has one primary hospital, one public health centre, and four private clinics. Since April 2016, cervical cancer screening service and treatment for pre cervical lesion has been offered to women aged 30 to 49 years. For this study, all women aged 30–49 years living in Finote Selam town were the source population.

### Sample size and sampling procedure

The sample size was determined using single population proportion formula (n = (Zά/2)^2^ p (1-p)/d^2^) with the following assumptions: 44.6% proportion of sufficient knowledge on cervical cancer, and 42.1% favourable attitude towards screening on the bases of a previous study done in Dessie, Ethiopia [11], A 95% level of confidence, 5 and 4% degree of precision for knowledge, and attitude respectively, two design effect, and 5% none response rate were used. The sample sizes calculated were 797 and 1224 for knowledge and attitude, respectively. Sample size was also calculated for factors using EPI info version 7 considering a 95% level of confidence, and 80% power. However, the sample size for factors were found less than the sample size calculated for the outcome variables (knowledge and attitude). So, we considered 1224 as sample size for this study.

### Operational definitions

#### Having comprehensive knowledge on cervical cancer

Refers to women who answered mean value and above of the twenty knowledge questions [15].

#### Favourable attitude towards cervical cancer screening

refers to women who answered mean and above of the six attitude questions Six attitude questions were used with five likert scale measurement: strongly disagree, disagree, neither agree nor disagree, agree, and strongly agree. A woman who scored mean value and above was considered as having a favourable attitude.

### Data collection instrument and process

The data collection tool (questionnaire) was developed by reviewing literature [11, 15–17]. The questionnaire was first prepared in English and translated to Amharic then back to English to ensure consistency. The questionnaire carried socio-demographic characteristics, risk of exposure to cervical cancer and reproductive health service utilization, knowledge and attitude assessing questions. The attitude questions were developed using a five level likert scale. Six female diploma and two BSc degree graduated midwives were recruited for data collection, and supervision, respectively. Face-to-face interviewer administered questionnaire was used.

To ensure data quality, a two day training was given to data collectors and supervisors. The questionnaire was pre-tested on 42 women who were living outside the selected kebeles to check the response, the clarity, and the appropriateness of the questions. The data collection and supervision were overseen by researchers to ensure completeness and consistency of data.

### Data analysis

The data were checked for completeness, coded manually, and entered into EPI-info version 7 and transferred to SPSS version 20 for analysis. Descriptive statistics were expressed in numerical value, mean, standard deviation, and percentages. Both bivariate and multivariable logistic regression analyses were done to identify variables associated with knowledge on cervical cancer, and attitude towards screening.Variables with less than 0.2 *P*-value, in the bivariate analysis were entered into multivariable analysis to control potential confounders. Hosmer-Lemeshow goodness of fit test was used to check the model fitness. Adjusted odds ratio with 95% confidence interval was used to determine the presence, degree, and direction of association between covariates and the outcome variable.

## Results

### Socio demographic characteristics participants

A total of 1137 women participated in this study giving a response rate of 93.7%. Ten questionnaires were incomplete. The mean age of the participants was 37.4 years (SD+ 5.72 years). The majority (92.2%) of study participants were Orthodox Christians, two-thirds (66.4%) of the women were married, and nearly half 513(45.1%) had no formal education. Out of the total participants, 530(46.6%) reported that their main occupation was household activities. One quarter (24.5%) of the participants had less than 23 US$ family average monthly income, and 37.7% had more than 68 US $ (Table 1).

**Table 1:** Socio-demographic characteristics of study participants in Finote Selam town, northwest Ethiopia, 2017

Characteristics	Frequency *n* = 1137(%)	Compressive knowledge		*P*-value	Attitude		*P*-value
		No	Yes		No	Yes	
Age							
30–34	372(32.7)	249	123	0.003	163	209	0.142
35–39	366(32.2)	241	125	0.001	130	236	0.001
40–44	200(17.6	145	55	0.137	83	117	0.80
45–49	199(17.5)	157	42		100	99	
Religion							
Orthodox	1048(92.2)	737	351		449	599	
Muslim	81(7.1)	51	30	0.16	25	56	0.037
Protestant	8(0.7)	4	4	0.22	2	6	0.322
Marital status							
Married	755(66.6)	29	28	0.001	13	44	0.001
Divorced	221(19.4)	523	232	0.73	301	454	0.50
Windowed	104(9.2)	159	62	0.257	110	111	0.970
Single	57(5)	81	23		52	52	
Educational status							
No formal education	513(45.1)	455	58		276	237	
Primary school	295(26)	220	75	.000	124	171	.001
Secondary school (9–12)	198(14)	81	117	.000	53	145	.000
College/university	131(11.5)	36	95	.000	23	108	.000
Occupation							
Household activities	530(46.)	434	96		270	260	
Self employed	254(22.3)	166	88	.000	87	167	0.000
Farmerand daily worker	169(15)	132	37	0.27	74	95	0.11
Government employed	98(8.6)	22	76	0.000	16	82	0.000
Private employee	73(6.4	31	42	0.000	27	46	0.027
Student	13(1.1)	7	6	0.017	2	11	0.024
Family average monthly income^a^							
< 22	278(24.5)	234	61		142	153	
22–45	344(30.3)	288	79	0.276	171	196	0.693
45–68	85(7.5)	47	24	.020	30	41	0.373
> 68	430(37.7)	223	181	.000	133	271	.000

^a^in USD

### Women’s risk of exposure and reproductive health services utilization

More than half of the women, 615 (54.1%), had the first sexual intercourse at the age of 16 or below. The mean age of the first sexual intercourse of the women was 16.4 years (SD ± 3.29 years). More than two-thirds of participants, 788(69.3%), had two or more sexual partners in their life time. One thousand sixteen (89.4%) women had history of at least one pregnancy and 1002 (88%) history of at least one child birth. Among women who had history of pregnancy, 579 (57%) had antenatal follow up during their last pregnancy. Out of the total participants 163(14.3%) had at least one history of abortion in their life time. Approximately three-fourths of the women, 830(73%) used modern contraceptive in their life time, and 14.6% had history of oral contraceptive use. During data collection, 590(51.9%) were using modern contraceptive. Only 49 (4.3%) of the women reported that they had history of sexually transmitted disease.(Table 2) Sixty-four (5.6%) participants reported that they knew persons with cervical cancer.

**Table 2:** Women’s risk of exposure to cervical cancer and reproductive health services utilization in Finote Selam town, northwest Ethiopia

Characteristics		Frequency	Percent
Age at first sexual intercourse	<=16 years > 16 years	615 522	54.1 45.9
Life time number of sexual partners	One Two and more	349 788	30.7 69.3
History of pregnancy	Yes No	1016 121	89.4 10.6
History of child birth	Yes No	1002 135	88 12
ANC during the last pregnancy(*N* = 1016)	Yes No	579 437	57 43
Abortion experience during the life time	Yes No	163 974	14.3 85.7
PNC after the last child birth (*N* = 1002)	Yes No	259 743	25.8 74.2
Ever used modern contraceptive method in the life time	Yes No	830 307	73 27
Modern contraceptive use during data collection	Yes No	590 547	51.9 48.1
Self reported history of STI during life time	Yes No	49 1088	4.3 95.7
Knew person with cervical cancer	Yes No	62 1075	5.6 94.4

*Abbreviation*: *ANC* Ante Natal Care, *PNC* Post Natal Care, *STI* Sexually Transmitted Infections

### Knowledge about cervical cancer

The awareness of the women on cervical cancer was 34.3% (95%CI: 31.7, 36.9). The major source of information was the mass media for 163(41.7%), family, friends, and neighbors for 160(41%). The number of the participants who listed at least one symptom of cervical cancer was 240 (21.1%) (95%CI: 18, 24). The most commonly mentioned symptom for cervical cancer was inter-menstrual bleeding 99 (8.7%). Most of the participants 897(78.9%) did not know any symptom of cervical cancer. Slightly less than a quarter, 264(23.2%) (95%CI: 19.6,26), of the participants identified at least one risk factor for the diseases. The most commonly mentioned, 138 (12.1%), risk factor for cervical cancer was multiple sexual partners.

**Fig. 1:**
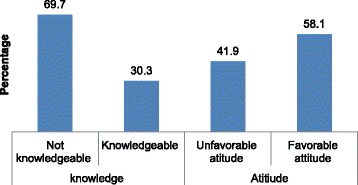
Comprehensive knowledge on cervical cancer and attitude towards screening among women aged 30–49 years in Finote Selam town, northwest Ethiopia, 2017

Out of participants, 259 (22.8%) (95%CI: 20, 27.3), mentioned at least one prevention methods of cervical cancer. Avoiding multiple sexual partners, 181 (15.9%), and early sexual intercourse, 60 (5.3%), were the most commonly mentioned ways of prevention. Nearly three-fourths participants, 866 (76.2%) reported that cervical cancer could not be treated even if diagnosed early stage. With regard to treatment of the diseases, surgery was the most frequently mentioned method by 175 (15.4%) followed by chemotherapy pointed out by 167 (14.7%) (Table 3). About 30.3% (95%CI: 27.7, 32.9) of the participants had comprehensive knowledge of cervical cancer (Fig. 1).

**Table 3:** Knowledge of participants of cervical cancer by components among aged 30-49 years women in Finote Selam town, northwest Ethiopia, 2017

Variables	Frequency *n* = 1137	Percent
Symptom of cervical cancer		
Inter menstrual bleeding	99	8.7
Foul smelling vaginal discharge	95	8.4
Pain during sexual intercourse	69	6.1
Vaginal bleeding during and after sexual intercourse	20	1.8
Risk factors of cervical cancer		
Multiple sexual partners	138	12.1
Early sexual intercourse	67	5.9
HIV infection	43	3.8
HPV infection	40	3.5
Others	4	0.4
Prevention of cervical cancer		
Avoiding multiple sexual partners	181	15.9
Avoiding early sexual intercourse	60	5.3
Avoiding pregnancies	24	2.1
Screening for pre-cervical cancer lesions	22	1.9
Vaccination of HPV	4	0.4
Cervical cancer can be treated if diagnosed early stage	271	23.8
Treatment for cervical cancer		
Surgery	175	15.4
Chemotherapy	167	14.7
Radiotherapy	15	1.3

*Abbreviation*: *HPV* human papiloma virus

Note: Others = multiparty and oral contraceptive use

### Women’s attitude towards cervical cancer screening

The majority of the women, 1046 (92%), agreed on consulting a health care professional during inter- menstrual bleeding. About half of the participants, 586 (51.5%), agreed that any sexually active woman is at risk of acquiring cervical cancer, and 429(37.8%) participants agreed that cervical cancer could be transmitted sexually. Two-thirds 744 (65.4%) agreed that pre-cervical cancer lesion screening helped to prevent cervical cancer. Among respondents, 699 (61.5%) stated that pre cervical cancer screening was not harmful. Significant proportions of women (87%) agreed to screen for the future if screening was free of charge. The proportion of women who had favorable attitude towards cervical cancer screening was 58.1% (95% CI: 55, 62.2) (Fig. 1).

### Factors associated with women’s comprehensive knowledge on cervical cancer

The bivariate analysis showed that socio-demographic variables, such as age, marital status, education, occupation, average family monthly income, knowing anyone who had cervical cancer, and reproductive history including number of life-time sexual partner, gravidity, ANC service utilization, parity, PNC service utilization, ever use of modern contraceptives, currently using contraceptives and history of STD were significantly associated with the comprehensive knowledge score of cervical cancer at *P*-value of less than 0.2. In the multi variable analysis, educational status, knowing someone with cervical cancer, and history of STD remained significant.

Participants who attended primary school were nearly two times (AOR = 1.65, 95% CI: 1.05, 2.59) more likely to be knowledgeable on cervical cancer than those who did not attend formal education. Women who attend secondary school (grade 9–12) were more than five times (AOR = 5.59, 95% CI: 3.25, 9.63) more likely to be knowledgeable than those who did not have formal education. Study participants who attended college/university were seven times (AOR = 7.21,95%CI: 3.41,15.29) more likely to be knowledgeable than those who did not have formal education. Women who knew someone with cervical cancer were five times (AOR = 5.38, 95% CI: 2.38, 12.15) more likely to be knowledgeable on cervical cancer compared to those who did not know cancer cases.

In the present study, women who had history of sexually transmitted diseases (STD) were nearly three times (AOR = 2.75, 95%CI: 1.24, 6.04) more likely to be knowledgeable than those who had no history of STD (Table 4).

**Table 4:** Bivariate and multivariable logistic regression analysis of factor associated with knowledge on cervical cancer among women aged 30–49 years Finote Selam town, northwest Ethiopia, 2017

Factors	Knowledge on cervical cancer		COR	95% CI	AOR	95%CI
	Not knowledgeable	Knowledgeable				
Age						
30–34	249 (66.9)	123 (33.1)	1.85	1.23–2.76^**^	1.27	0.72–2.43
35–39	241 (65.8)	125 (34.2)	1.94	1.29–2.90^**^	1.40	0.76–2.59
40–44	145 (72.5)	55(32.5)	1.42	0.89–2.24	1.33	0.68–2.59
45–49	157 (78.9)	42(21.1)	1		1	
Marital status						
Single	29(51)	28(49)	3.40	1.69–9.63^*^	0**.**92	0.32–2.62
Married	523 (69.3)	232(30.7)	1.56	0.95–2.54	0.81	0.38–1.72
Divorced	159 (72)	62(28)	1.37	0.79–2.37	0.94	0.42–2.12
Windowed	81 (77.8)	23(22.2)	1		1	
Educational status						
No formal education	455 (88.6)	58(11.4)	1		1	
Primary	220 (74.5)	75(25.5)	2.67	1.83–3.90^***^	1.65	1.04–2.59^**^
Secondary (9_12)	81 (40.9)	117(59.1)	11.33	7.64–16.79^***^	5.6	3.25–9.63^***^
College/university	36 (27.4)	95(72.6)	20.70	12.92–33.13^***^	7.21	3.41–15.28^***^
Occupation						
Home maker	434 (81.8)	96(18.2)	1		1	
Employed	53 (30.9)	118(69.1)	10.06	6.79–14.90^***^	1.66	0.89–3.08
Self employed	166 (65.3)	88(34.7)	2.40	1.70–3.36^***^	1.04	0.67–1.63
Farmer, daily labor	132 (78.1)	37(21.9)	1.26	0.87–1.94	1.37	0.81–2.33
Student	7 5 (53.8)	6(46.2)	13.87	1.27–11.78^**^	1.04	0.27–4.02
Family monthly income^a^						
< 22	234 (79.3)	61(20.7)	1		1	
22–45	288 (78.4)	79(21.6)	1.27	0.83–2.24	0.73	0.42–1.15
45–68	47 (66.1)	24(33.9)	1.96	1.11–3.45^*^	0.70	0.30–1.64
> 68	223 (55.1)	181(44.9)	3.11	2.20–4.38^***^	0.97	0.38–1.67
Knowing anyone who had Cervical cancer						
No	775 (70.3)	298(29.7)	1		1	
Yes	17 (26.5)	47(73.5)	7.19	4.06–12.72^***^	5.38	2.38–12.2^***^
No of sexual partners						
One	218 (62.4)	131(37.6)	1		1	
More than one	574 (72.8)	214(27.2)	1.61	1.23–2.10^**^	1.38	0.95–2.06
Gravidity						
0	68 (56.1)	53(43.9)	1		1	
1	73 (58.8)	51(41.2)	0.89	0.54–1.48	1.35	0.28–6.36
2–4	460 (60.6)	200(39.4)	0.56	0.37–0.82^**^	1.02	0.22–4.77
> =5	191 (82.3)	41(17.7)	0.28	0.16–0.45^***^	2.07	0.23–18.20
Parity						
Null-porous	79 (58.5)	56(41.5)	1		1	
1	88 (58.6)	62(41.4)	099	0.62–1.59	0.96	0.21–4.37
2–4	444 (70.1)	189(29.9)	0.60	0.41–0.88^**^	0.89	0.19–4.14
> =5	181 (82.6)	38 17.4)	0.30	0.18–0.48^**^	0.53	0.06–1.78
ANC utilization for last pregnancy						
No	426 (76.80	128(23.2)	1		1	
Yes	366 (62.7)	217(37.3)	1.97	1.52–2.55^***^	1.24	0.79–1.93
PNC utilization for last delivery						
No	642 (73.1)	236(26.9)	1		1	
Yes	150 (57.9)	109(42.1)	1.98	1.48–2.63^***^	1.19	0.80–1.78
Ever-use modern contraceptive						
No	263 (85.6)	44(14.4)	1		1	
Yes	529 (63.7)	301(36.3)	3.40	2.39–4.82^***^	6.54	0.54–87.64
Currently using contraceptive						
No	274 (68.6)	125(31.4)	1		1	
Yes	254 (58.9)	177(41.1)	1.57	1.14–2.03^**^	1.08	0.76–1.54
History of STD						
No	774 (71.1)	314(28.9)	1		1	
Yes	18 (36.7)	31(63..3)	4.5	2.34–7.70^***^	2.75	1.24–6.04^**^

^a^in USD, *COR* Crude odds ratio, *AOR* adjusted odds ratio, *CI* Confidence interval, *ANC* Ante-Natal care, *PNC* Post-Natal care, *USA* United States of America, 1: Reference category, *STD* Sexually transmitted disease, ^*^:0.05 < *p* < 0.2, ^**^:0.001 < *p* < 0.05,^***^:*p* < 0.001

### Factors associated with a ttitude towards cervical cancer screening

According to the bivariate analysis, seven socio-demographic variables, age, religion, marital status, education, occupation, average family monthly income, knowing someone with cervical cancer, including nine other variables were significantly associated with attitude to cervical cancer screening at *P*-value of less than 0.2. In the multivariable analysis, three variables, such as educational status, knowing someone with cervical cancer and comprehensive knowledge about the diseases became significant with attitude towards cervical cancer screening at a P-value of less than 0.05.

Participants who attended college/university were 2.56 times (AOR = 2.56,95%CI:1.14,5.69) more likely to have a favorable attitude towards screening than those who did not attend formal education. Women who knew someone who had cervical cancer were three times (AOR = 3.24, 95% CI: 1.14, 9.15) more likely to have a favorable attitude towards cervical cancer screening. Women who had comprehensive knowledge of cervical cancer were three times (AOR = 3.00, 95%CI: 1.97, 4.29) more likely to have a favorable attitude towards cervical cancer screening than their counterparts (Table 5).

**Table 5:** Bivariate and multivariable analysis of factor associated with attitude towards cervical cancer screening among women aged 30–49 years Finote Selam town, northwest Ethiopia, 2017

Factors	Attitude towards screening		COR	95% CI	AOR	95%CI
	Unfavorable attitude	Favorable attitude				
Age						
30–34	163 (43.8)	209 (56.2)	1.29	0.91–1.82^*^	0.98	0.68–2.10
35–39	130 (35.5)	236 (64.5)	1.83	1.29–2.60^**^	0.73	0.42–1.27
40–44	83 (41.5)	117 (58.5)	1.42	0.95–2.11^*^	0.83	0.45–1.50
45–49	100 (50.2)	99 (49.8)	1		1	
Religion						
orthodox	449 (42.8)	599 (57.2)	1		1	
Muslim	25 (30.2	56 (69.8)	1.80	1.03–2.73^*^	0.74	0.40–1.50
Protestant	2 (25)	6 (75)	0.74	0.14–3.96	0.36	0.05–2.22
Marital status						
Single	13 (22.8)	44 (77.2)	3.38	1.63–7.01^**^	2.08	0.74–5.87
Married	301(39.8)	454 (60.2)	1.50	1.00–2.27^*^	1.07	0.53–2.13
Divorced	110(49.7)	111 (52.3)	1.01	0.63–1.60	0.63	0.30–1.31
Windowed	52 (50)	52 (50)	1		1	
Education						
No formal education	276 (53.80)	237 (46.2)	1		1	
Primary	124 (42)	171 (58)	1.61	1.20–2.14^**^	1.32	0.90–1.95
Secondary (9_12)	53 (26.7)	145 (73.3)	3.16	2.22–4.56^***^	1.54	0.88–2.67
College/university	23 (17.5)	108 (82.5)	5.46	3.37–8.85^***^	2.56	1.14–5.69^**^
Occupation						
Home maker	270 (50.9)	260 (49.1)	1		1	
Employed	43 (25.1)	128 (74.9)	3.09	2.10–4.54^***^	0.74	0.38–1.44
Self employed	87(34.2)	167 (65.8)	2	1.46–2.71^***^	1.30	0.84–2.02
Farmer & daily labor	74 (43.7)	95 (56.3)	1.33	0.94–1.88^*^	1.67	0.94–2.67
Student	2 (15.3)	11 (84.7)	5.71	1.25–26.01^**^	1.7	0.32–9.00
Family average monthly income^a^						
< 22	142 (47.9)	153 (52.1)	1		1	
22–45	171 (46.5)	196 (53.5)	1.06	0.78–1.44	0.94	0.61–1.46
45–68	30 (42.2)	41 (57.8)	1.26	0.75–2.14	0.70	0.33–1.48
> 68	133 (32.9)	271 (67.1)	1.9	1.38–2.57^***^	1.14	0.69–1.89
Knowing anyone who had CCa						
No	464 (43.2)	609 (56.8)	1		1	
Yes	12 (18.7)	52 (81.3)	3.30	1.74–6.25^***^	3.24	1.14–9.15^**^
No of sexual partners						
One	127 (36.3)	222 (63.7)	1		1	
More than one	349 (44.2)	439 (55.8)	0.72	0.55–0.93^**^	1.03	0.71–1.49
Gravida						
0	43 (35.5)	78 (64.5)	1		1	
1	51 (41.1)	73 (58.9)	0.78	0.47–1.32	1.10	0.26–4.61
2–4	264 (40)	396 (60)	0.82	0.53–1.23	1.29	0.29–5.74
> =5	118(50.8)	114 (49.2)	0.533	0.33–0.83^**^	1.70	0.19–14.63
ANC service utilization						
No	270 (48.7)	284 (51.3)	1		1	
Yes	206(35.3)	377 (64.7)	1.74	1.37–2.20^***^	1.07	0.72–1.57
Parity						
Nulparous	48 (35.5)	87 (64.5)	1		1	
1	62 (41.3)	88 (58.7)	1.93	1.24–3.00^**^	0.76	0.19–3.03
2–4	253 (39.9)	380 (60.1)	1.51	0.95–2.30^*^	0.78	0.18–3.38
> =5	113 (51.5)	106 (48.5)	1.60	1.17–2.18^**^	0.55	0.06–4.65
PNC service utilization for last delivery						
No	403 (48.5)	475 (52.5)	1		1	
Yes	73 (28.1)	186 (71.9)	2.16	1.59–2.92^***^	1.43	0.97–2.10
Ever use modern contraceptive						
No	172 (56)	135 (44)	1		1	
Yes	304 (36)	526 (64)	2.20	1.69–2.87^***^	2.00	0.44–9.07
Currently using contraceptive						
No	156 (39)	243 (61)	1		1	
Yes	149 (34.5)	282 (65.5)	1.52	1.14–2.03^*^	0.95	0.68–1.32
History of STD						
No	464 (42.6)	624 (37.4)	1		1	
yes	12 (24.4)	37 (75.6)	2.29	1.18–4.44^**^	1.27	0.55–2.93
Knowledge on Cervical cancer						
Not knowledgeable	407 (51)	385 (49)	1		1	
Knowledgeable	69 (20)	276 (80)	4.23	3.13–5.69^***^	3.00	1.97–4.29^***^

^a^in USD, *CCa* Cervical cancer, *COR* Crude odds ratio, *CI* Confidence interval, *AOR* Adjusted Odds Ratio, *ANC* Ante-Natal care, *PNC* Post-Natal care, *USA* United States of America,1: Reference category, *STD* Sexually transmitted disease,^*^:0.05 < *p* < 0.2, ^**^:0.001 < *p* < 0.05,^***^:*p* < 0.001

## Discussion

Knowledge of cervical cancer and attitude towards its screening are essential for the prevention of the disease. The current study was conducted to determine women’s comprehensive knowledge on cervical cancer, attitude towards pre-cervical cancer screening and associated factors.

The study revealed that nearly one-third, 30.3% (95%CI: 27.2–32.9), of participants had comprehensive knowledge on cervical cancer. This finding is comparable with that of a community based study conducted in Gondar (31%), Ethiopia, among women aged 15 years and above [15]. This finding is higher than the finding 25% reported from Addis Ababa [18]. The possible reason for difference might be that the researchers used only 13 questions and 7/13 was used as the cutoff point to determine knowledge in the previous study. However, our result is lower than the finding reported by a community-based study in Uganda which noted 55% [19]. The possible reason for the difference from the study done in Uganda could be the variation in educational status between the two study populations. The majority of the participants in the Ugandan study completed primary school unlike ours in which nearly half of participants did not have formal education.

Ours study showed that 58.1% (95% CI: 55, 62.2) of the women had favourable attitude towards cervical cancer screening. This result was better than that of a study done in Dessie, Ethiopia which documented 42.1% [11]. The gap between our study and that of Dessie might be the high proportion of single (unmarried) participants in the Dessie study. There is evidence that single women have less favourable attitude towards screening [16]. However, the result is lower than the finding of a hospital-based study in India which reported 76% [16]. The difference between our and the study in India might be the study setting; hospitalized women might have high health seeking behaviour and better access to information. Our finding was also lower than that of a study conducted among market women in Nigeria noted 80.4% had favourable attitude towards screening [20]. The difference might be that in the Nigeria study the participants were shop owners/attendants aged 15 years and above, most of them with higher educational status.

In this study, educational status was positively associated with women’s comprehensive knowledge on cervical cancer. Women who have primary, secondary and college/university education were more likely to have better knowledge on cervical cancer than those who did not have formal education. Studies in the Democratic Republic of Congo [10], India [16], and Dessie, Ethiopia [11], reported similar findings. Similarly, in this study, it was found that educational status of women had a significant association with attitude towards cervical cancer screening. In the present study, women who had college and above educational status were more than two times more likely to have favorable attitude towards cervical cancer screening. Similar results were found by studies conducted in Eastern Uganda [19], and Addis Ababa [18].

The present study identified that the participants who knew someone with cervical cancer were five times more likely to be knowledgeable on cervical cancer than those who didn’t know someone with cervical cancer. Studies conducted in Gondar [15], and Kenya [21] had similar findings. Our study revealed that women who knew someone who had cancer were 3.24 times more likely to have favorable attitude towards cervical cancer screening (AOR = 3.24, 95%CI: 1.14, 9.15).

Our finding showed that women who had history of STD were nearly three times more likely to be knowledgeable on cervical cancer than those who had not. A similar finding was reported by a study done on Asian Maldives [22]. This might be because of women who had STD might have contact with health professionals to get treatment for the disease during which they might be advised/counselled about the risks of unprotected sex, including the risk of viral infections, such as HIV, and the human papiloma virus plus their consequences and means of prevention. Thus, this communication might help them get information on cervical cancer. A study conducted in rural Kenya identified that family planning service utilization was positively associated with knowledge on cervical cancer [9]. In our study, no significant association was observed between knowledge on cervical cancer and obstetric service related variables such as ANC, family planning and post-natal service utilization. This may suggest that women who utilize maternal health services do not receive information on cervical cancer.

The finding in this study showed that women who had comprehensive knowledge of cervical cancer were three times more likely to have favorable attitude towards cervical cancer screening as compared to their counterparts. Previous studies done in Nepal [23], and a health facility-based study in Dessie Ethiopia [11], reported a similar finding. This shows that women who have adequate knowledge on cervical cancer might understand the nature disease (causes, symptoms, preventions, and treatments), and the benefits of screening which in turn make them have positive attitude towards screening. Since this study was conducted in urban setting that could not generalize for rural women. This might be the potential limitation of this study. Additionally, one of the independent variables we considered was history of sexually transmitted diseases that might be exposed for social desirability bias. However, the authors tried to minimize this potential bias by providing adequate information for the study participants about the importance of telling the truth and confidentiality nature of the research.

## Conclusion

In this study, it was identified that women’s knowledge on cervical cancer was low, despite the high incidence of the disease in Ethiopia. Relatively, a large proportion of the study participants in this study had favorable attitude towards cervical cancer screening. Attending primary, secondary school and college, knowing someone who had cervical cancer and history of STD were factors associated to comprehensive knowledge on cervical cancer.

Attended college and above, knowing someone who had cervical cancer, and having comprehensive knowledge on cervical cancer were important factors for having favorable attitude towards cervical cancer screening. There is clear need for information sharing on cervical cancer, including its screening targeting women with less educated. Educational campaign on the disease and screening,involving the mass media in providing information to the women on cervical cancer, including screening service availability when the women visit health facilities for reproductive services are proposed.

## Abbreviations

ANC: Ante Natal care
AOR: Adjusted Odds Ratio
CI: Confidence Interval
PNC: Post Natal care
SD: Standard Deviation
SPSS: Statistical Package for Social Sciences
STD: Sexually transmitted disease
